# Alanine-specific appetite in slow growing chickens is associated with impaired glucose transport and TCA cycle

**DOI:** 10.1186/s12864-022-08625-2

**Published:** 2022-05-23

**Authors:** Shahram Niknafs, Marina R. S. Fortes, Sungbo Cho, John L. Black, Eugeni Roura

**Affiliations:** 1 Centre for Nutrition and Food Sciences, Queensland Alliance for Agriculture and Food Innovation, The University of Queensland, St Lucia, QLD 4072 Australia; 2 School of Chemistry and Molecular Bioscience, The University of Queensland, St Lucia, QLD 4072 Australia; 3 John L Black Consulting, Warrimoo, NSW 2774 Australia

**Keywords:** Alanine, Chicken, Growth, Glucose, Pyruvate, Feed intake

## Abstract

**Background:**

The rate of protein accretion and growth affect amino acid requirements in young animals. Differences in amino acid metabolism contribute to individual variations in growth rate. This study aimed at determining how amino acid needs may change with growth rates in broiler chickens. Experiment 1 consisted of testing amino acid choices in two chicken groups with extreme growth rates (the slowest –SG- or fastest –FG- growing birds in a flock). Essential (EAA) (methionine, lysine and threonine) or non-essential (NEAA) (alanine, aspartic acid and asparagine) amino acids were added to a standard control feed (13.2 MJ/kg; 21.6% crude protein). The chickens were offered simultaneous access to the control feed and a feed supplemented with one of the two amino acid mixes added at 73% above standard dietary levels. Experiment 2 consisted of the selection of the bottom 5 SG and top 5 FG chickens from a flock of 580 to study differences in amino acid metabolism using the proventriculus representing gut sensing mechanism. In this experiment, transcriptomic, proteomic, and genomic analyses were used to compare the two groups of chickens.

**Results:**

SG preferred NEAA, while they rejected EAA supplemented feeds (*P* < 0.05). However, FG rejected NEAA (*P* < 0.05), and they were indifferent to EAA supplemented feed (*P* > 0.05). Transcriptomic and proteomic analyses identified 909 differentially expressed genes and 146 differentially abundant proteins associated with differences in growth rate (*P* < 0.05). The integration of gene expression and protein abundance patterns showed the downregulation of sensing and transport of alanine and glucose associated with increased alanine catabolism to pyruvate in SG chickens.

**Conclusion:**

Dietary preferences for NEAA in the SG group are associated with a potential cytosolic depletion of alanine following an upregulation of the catabolism into TCA cycle intermediates.

**Supplementary Information:**

The online version contains supplementary material available at 10.1186/s12864-022-08625-2.

## Background

Chickens have been used as a model organism for more than a century for research into embryology, immunology, genetics, cell biology, cancer, virology and growth [1–3]. Broiler chickens have been selected for efficient body weight gain for decades with modern breeds reaching one of the highest growth rates across livestock and laboratory animals. However, genetic selection has not been successful in improving growth uniformity within chicken populations.

Weight gain is strongly correlated to feed intake. Many factors contribute to feed intake regulation in chickens [4]. Imbalances in available amino acids to energy in the diet can depress or enhance feed intake [5]. The fastest growing (FG) chickens in a flock deposit more body protein and have higher amino acid requirements than the average. These birds increase feed intake to compensate for the increased need for amino acids [6–9]. In contrast, slow growing (SG) chickens consume excessive amino acids from the diet relative to the flock average, which can supress their appetite and decrease feed intake [10, 11]. Broiler feeds are formulated primarily to meet the requirements of the limiting essential amino acids (EAA) [7, 12]. In contrast, the supply of non-essential amino acids (NEAA) is rarely considered. The NEAA are considered to be synthesised in sufficient quantity from metabolic precursors within the animal’s cell. However, mounting evidence is building in the scientific literature showing that NEAA can stimulate feed intake and growth rate of chickens, particularly when offered in low protein diets [6, 13].

Nutrient sensing is an essential mechanism enabling broilers to select diets that balance their nutrient requirements, when given a choice of feeds varying in nutrient composition [14, 15]. The molecular mechanisms that mediate choice and specific appetites for amino acids in chickens have been related to the taste receptor dimer T1R1/T1R3, amongst other receptors [15, 16]. However, a systematic study on preferences between diets varying in amino acid concentrations has not been reported in broilers. Potential differences in amino acid sensing mechanisms may explain lower feed intake and slow growth in SG compared to FG chickens.

The objective of this research was to study differences in specific amino acid appetites in broilers with extreme high or low growth rates. The hypothesis tested was that specific amino acid appetites associated with low growth rates indicate innately constrained metabolic pathways affecting feed intake and protein accretion in SG broiler chickens.

## Results

The selection of the 10% lightest and heaviest individuals in a flock resulted in 29% higher (*P* < 0.05) final body weight (42 days) and feed intake in FG compared to SG chickens. Double choice (DC) treatments did not significantly (*P* > 0.05) affect growth or total feed intake during the experimental period. A summary of the main performance parameters and statistical significance of main effects have been presented in the Table S3 in Additional file 1.

### Double-choice studies

**Fig. 1:**
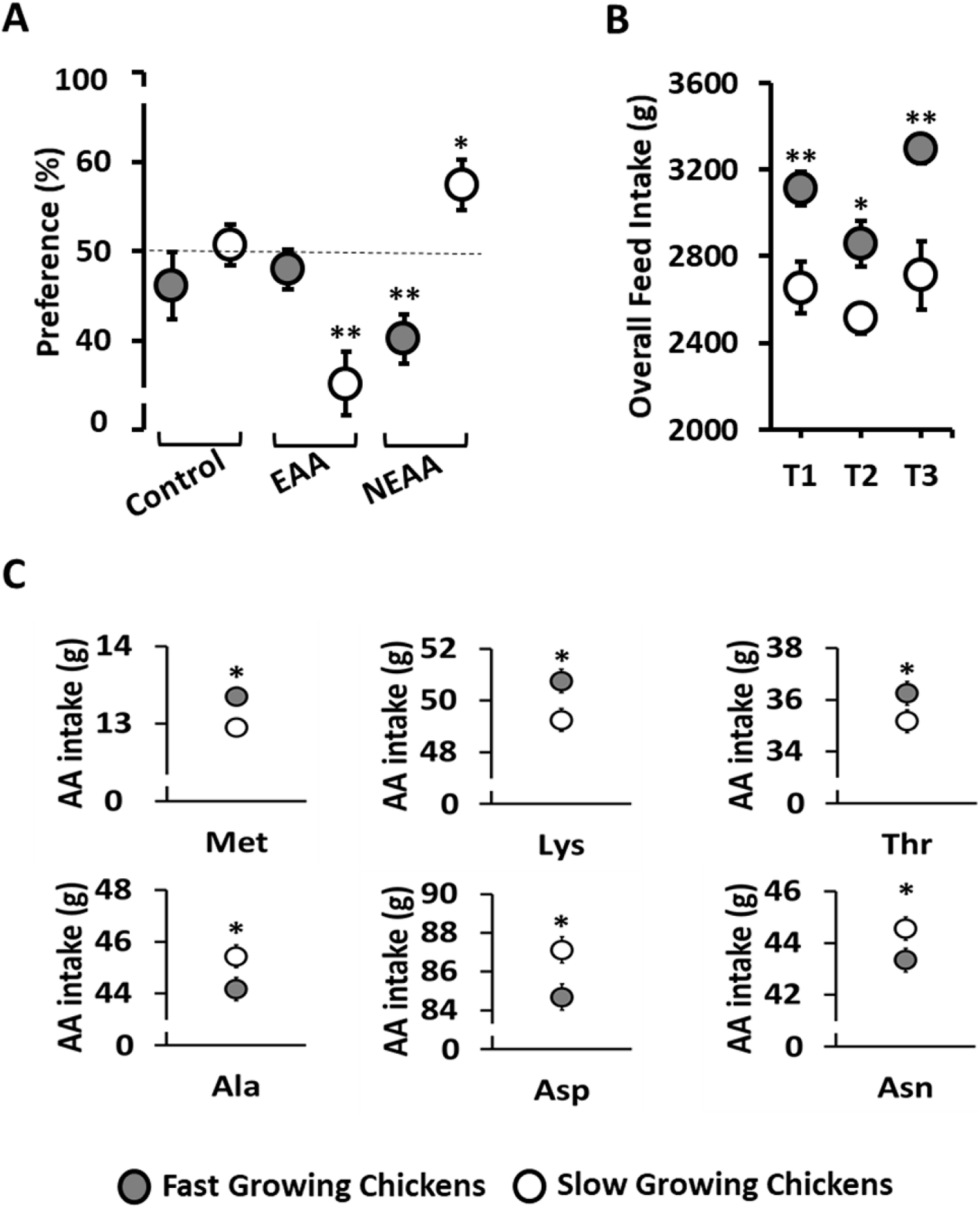
Comparison of preference and intake between slow (SG) and fast (FG) growing broiler chickens. Feed and amino acid intake and preference over two weeks in SG and FG in double-choice tests between commercial feed and feed supplemented with excess of a mix of essential amino acids (EAA: Met, Lys, and Thr) or non-essential amino acids (NEAA: Ala, Asp and Asn): **A** Comparison of preference values (percentage of test diet intake divided by the summation of test diet and control diet) for different groups of amino acids in SG and FG chickens; **B** Overall feed intake (adding the intake from both feeders) in SG compared to FG chickens; **C** Comparison of the amount of amino acids consumed in the preference test after adjusting for the covariate effect of total feed intake between SG and FG chickens. T1: feed vs feed (control); T2: feed vs feed+EAA; T3: feed vs feed+NEAA. One ^*^ or two ^**^ asterisks mean significant differences at *P* < 0.05 or *P* < 0.01 levels, respectively

The SG birds showed a significant (*P* < 0.05) preference for the NEAA (Ala, Asp, Asn) supplemented feed compared to the control feed (Fig. 1A). In contrast, SG chickens rejected (*P* < 0.01) the EAA (Met, Lys and Thr) supplemented feed compared to the control feed. The FG chickens significantly (*P* < 0.01) chose against the NEAA supplemented feed while showing no preference (*P* > 0.05) for the EAA supplemented feed (Fig. 1A).

FG birds consumed significantly (*P* < 0.01) more total feed (control + test feed) than SG chickens across all treatments (Fig. 1B). The analysis of covariance showed that SG chickens consumed significantly less (*P* < 0.05) EAA (Met, Lys, Thr), but more NEAA (Ala, Asp, Asn) than the FG birds after adjusting for total feed intake as a covariate (Fig. 1C).

### Gene and protein expression studies

**Fig. 2:**
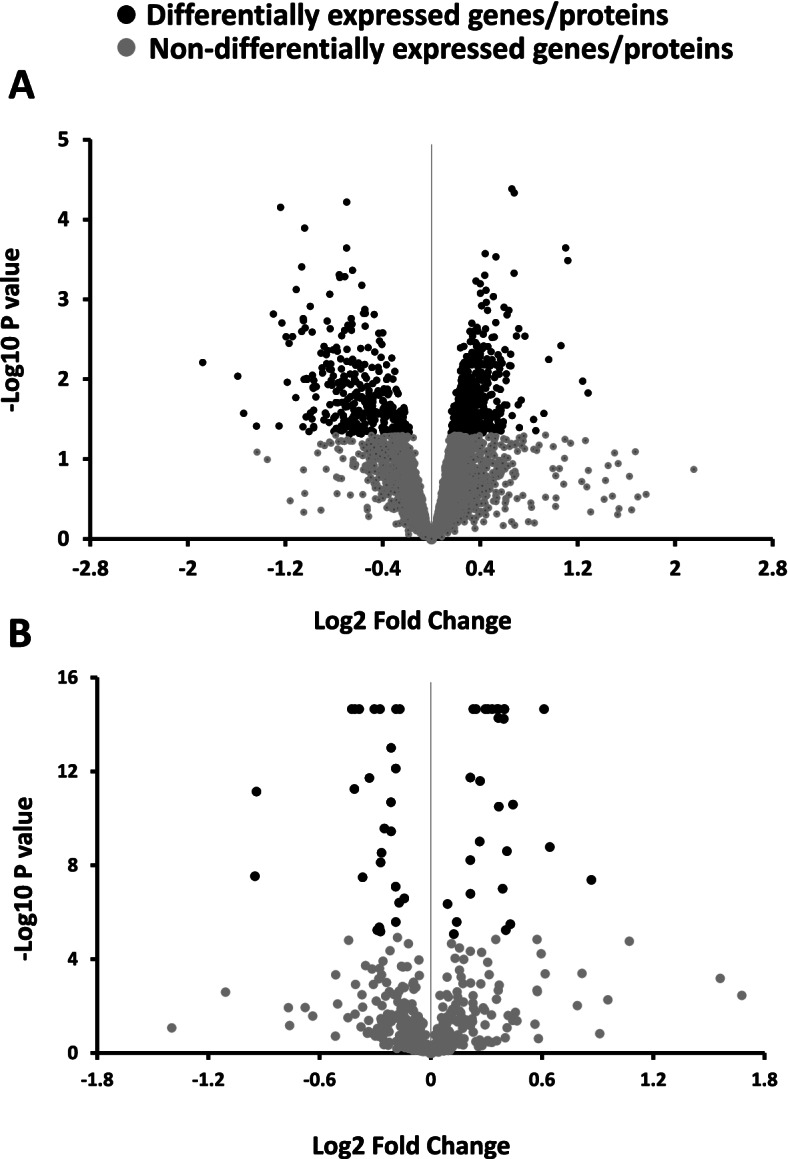
Volcano plot showing genes and proteins. **A** Differentially (DEG) and non-differentially (non-DEG) expressed genes; **B** differentially (DAP) and non-differentially (non-DAP) abundant proteins in the proventriculus of slow (SG) compared to fast (FG) growing chickens. Each dot represents a gene (**A**) or a protein (**B**). Black dots are differentially expressed genes or abundant proteins (*P* < 0.05), whereas grey dots show no difference between SG and FG chickens. Dots to the right of the vertical lines are downregulated, whereas the dots on the left are upregulated in SG chickens. There were 909 DEG and 146 DAP

RNA sequencing (RNAseq) analysis of the proventriculus found 909 DEG, and the proteomic analysis found 146 DAP when comparing SG to FG chickens (Fig. 2). The qPCR validation showed a high correlation coefficient (− 0.89; *P* < 0.0001) between gene expression of the reported genes measured using qPCR and RNAseq (Fig. S2 in Additional file 1). For example, both RNAseq and qPCR analyses showed that gene expression of α-gustducin was significantly down-regulated in SG compared to FG (Table S4 in Additional file 1).

**Fig. 3:**
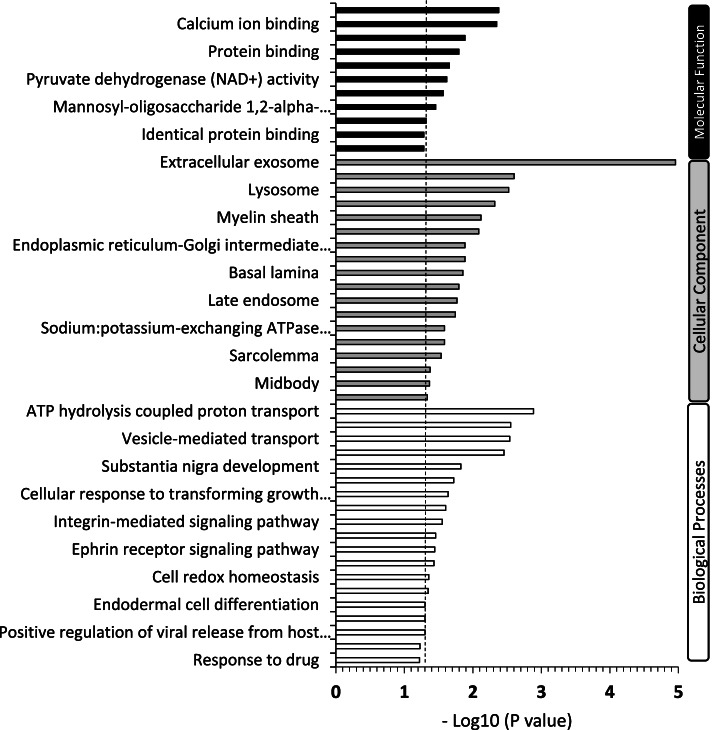
Gene Ontology (GO) enrichment analysis. GO shows direct terms (Biological Process, Cellular Component, Molecular Function) that were significantly enriched due to differentially expressed genes between slow (SG) vs fast (FG) growing chickens. The dash line represents the significant threshold at *P* < 0.05 level. Bars exceeding the threshold are significantly enriched

**Fig. 4:**
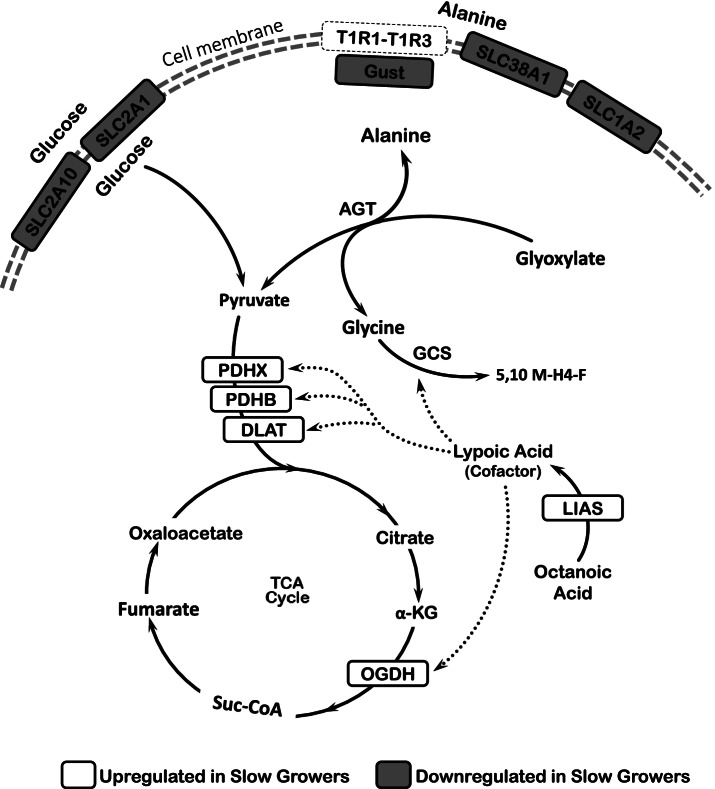
Integrated details of the enriched metabolic pathways from transcriptomic data. This represents up/down regulations of pathways (*P* < 0.05) in slow growing (SG) chickens. AGT: Alanine-glyoxylate transaminase; DLAT: dihydrolipoamide S-acetyltransferase; GCS: Glycine cleavage system; Gust: Gustducin (subunit alpha); LIAS: Lipoic acid synthetase; OGDH: oxoglutarate (α-ketoglutarate) dehydrogenase; PDHB: pyruvate dehydrogenase (lipoamide) beta; PDHX: pyruvate dehydrogenase complex component; SLC2A1, 10: Solute carrier 2 trype A1 and 10; SLC38A1: Solute Carrier 38 A1; SLC1A2: Solute Carrier 1 A2; T1R1-T1R3: Umami taste receptor

The DEG significantly (*P* < 0.05) enriched several biological processes, cellular components and molecular functions as shown in Fig. 3 (Gene Ontology). In addition, the results showed several enriched metabolic pathways including the ‘Glyoxylate Metabolism and Glycine Degradation’ and ‘Pyruvate Metabolism’ pathways (KEGG and REACTOME; Table 1). Five DEG (DLAT: dihydrolipoamide S-acetyltransferase; LIAS: Lipoic acid synthetase; OGDH: oxoglutarate (α-ketoglutarate) dehydrogenase; PDHB: pyruvate dehydrogenase beta; PDHX: pyruvate dehydrogenase complex component) relevant to the two pathways were significantly upregulated in SG chickens (Table 2, Fig. 4). In contrast, gene expression for several amino acid sensors and transporters including Gust (Gustducin, subunit alpha), SLC38A1 (Solute carrier 38 type A1), SLC1A2 (Solute carrier 1 type A2), and the glucose transporters SLC2A1 (Solute carrier 2 type A1) and SLC2A10 (Solute carrier 2 type A10) were downregulated in SG chickens (Fig. 4). The corresponding protein names of the genes SLC38A1, SLC1A2, SLC2A1, and SLC2A10 are SNAT1, EAAT2, GLUT1, and GLUT10, respectively.

**Table 1:** Significantly (*P* < 0.05) enriched metabolic pathways in KEGG and REACTOME. Differentially expressed genes (DEG) and differentially abundant proteins (DAP) involved in each pathway resulted from transcriptomic and proteomic analyses in proventriculus of fast (FG) vs slow (SG) growing chickens

Experiment	Enriched Biological Pathway	Upregulated DEG or DAP		Database^**a**^	***P*** value
		Upregulated in SG	Upregulated in FG		
Transcriptomic analysis	Glyoxylate Metabolism and Gly Degradation	DLAT, PDHB, PDHX, LIAS, OGDH	none	REACTOME	0.014
	Pyruvate Metabolism	DLAT, PDHB, PDHX	none	REACTOME	0.030
Proteomic analysis	Translocation of GLUT4 to membrane	none	YWHAE, YWHAQ, YWHAZ	REACTOME	0.016
	The Tricarboxylic Acid Cycle	SDHA, SDHB	CS	REACTOME	0.001
	Glycolysis	TPI1	ENO1, PKM	REACTOME	0.029
	Gluconeogenesis	GOT1, TPI1	ENO1	REACTOME	0.046
	Biosynthesis of Amino Acid	GOT1, PKM, TPI1	CS, ENO1, PRPS2	KEGG	0.003
	Arg and Pro Metabolism	none	AGMAT, CKB, CKM, CKMT1A, GOT1	KEGG	0.005

Abbreviations: *AGMAT* Agmatinase, *Arg* arginine, *CKB M* Creatine Kinase Brain, Muscle, *CS* Citrate synthase, *DAP* Differentially abundant proteins, *DEG* Differentially expressed genes, *DLAT* dihydrolipoamide S-acetyltransferase, *ENO1* Enolase 1, *GLUT4* glucose transporter 4, *Gly* glycine, *GOT1* Glutamic-oxaloacetic transaminase 1, *LIAS* Lipoic acid synthetase, *OGDH* oxoglutarate (α-ketoglutarate) dehydrogenase, *PDHB* pyruvate dehydrogenase (lipoamide) beta, *PDHX* pyruvate dehydrogenase complex component, *PKM* Pyruvate Kinase, *Pro* proline, *PRPS2* Phosphoribosyl pyrophosphate synthetase 2, *SDHA, B* Succinate dehydrogenase complex flavoprotein subunit A, B, *TPI1* Triosephosphate isomerase 1, *YWHAE, Q, Z* Tyrosine 3-monfooxygenase/ tryptophan 5-monooxygenase activation protein epsilon, theta, zeta

^a^The reference for the databases used in the study are: KEGG (Kanehisa and Goto, 2000), REACTOME (Lewis et al., 2005)

**Table 2:** Gene expression and protein abundance. Genes/proteins involved in the significantly (*P* < 0.05) enriched pathways comparing the expression levels between slow (SG) to fast (FG) growing chickens (*n* = 5)

Measurement	Identification of genes and proteins	SG Chickens	FG Chickens	SE	*P* value
Normalized Gene Expression^a^	DLAT	29.42	23.59	1.32	0.0152
	PDHX	6.11	5.12	0.23	0.0213
	OGDH	59.74	49.22	2.48	0.0222
	LIAS	9.53	8.49	0.27	0.0385
	PDHB	58.91	49.82	2.32	0.0402
Normalized Protein Abundance (× 10^6^)^b^	CKMT1A	4.22	3.37	0.41	0.0000
	CKM	1.51	0.94	0.14	0.0000
	CKB	13.21	8.72	1.11	0.0000
	CS	0.46	0.59	0.05	0.0000
	YWHAE	0.27	0.34	0.04	0.0028
	YWHAZ	0.97	1.11	0.12	0.0028
	SDHB	1.12	0.78	0.10	0.0044
	YWHAQ	0.10	0.13	0.02	0.0047
	TPI1	2.47	1.78	0.19	0.0051
	GOT1	1.21	0.86	0.10	0.0077
	PRPS2	0.08	0.09	0.01	0.0084
	ENO1	1.67	2.10	0.15	0.0126
	PKM	1.65	2.05	0.20	0.0335
	AGMAT	0.06	0.05	0.01	0.0350
	SDHA	0.50	0.36	0.05	0.0373

Abbreviations: *AGMAT* Agmatinase, *CKB, M* Creatine Kinase Brain, Muscle, *CS* Citrate synthase, *DAP* Differentially abundant proteins, *DEG* Differentially expressed genes, *DLAT* dihydrolipoamide S-acetyltransferase, *ENO1* Enolase 1, *GOT1* Glutamic-oxaloacetic transaminase 1, *LIAS* Lipoic acid synthetase, *OGDH* oxoglutarate (α-ketoglutarate) dehydrogenase, *PDHB* pyruvate dehydrogenase (lipoamide) beta, *PDHX* pyruvate dehydrogenase complex component, *PKM* Pyruvate Kinase, *PRPS2* Phosphoribosyl pyrophosphate synthetase 2, *SDHA, B* Succinate dehydrogenase complex flavoprotein subunit A, B, *SE* standard error, *TPI1* Triosephosphate isomerase 1, *YWHAE, Q, Z* Tyrosine 3-monooxygenase/ tryptophan 5-monooxygenase activation protein epsilon, theta, zeta

^a^Normalized gene expression refers to FPKM (Fragments Per Kilobase of transcript per Million mapped reads) calculated using RNA-seq data in Limma. Baysian model with t-statistics moderated across genes were used to identify significant differences. RNA-seq data were based on 2 × 125 bp Paired-End Dual indexed reads and average of ~ 27 million sequence reads per sample

^b^Normalized protein abundance refers to the peak area (intensity × retention time) of the proteins’ spectra. Peak areas were obtained using SWATH (Sequential Window Acquisition of All Theoretical Mass Spectra) analyses of LC-MS/MS data

**Fig. 5:**
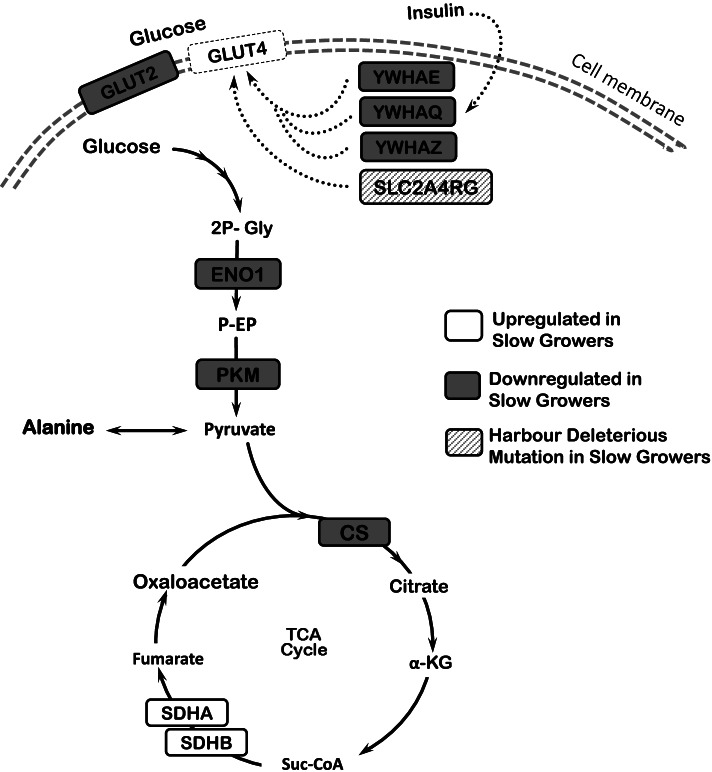
Integrated details of the enriched metabolic pathways from proteomic data as well as genomic analysis. Figure represents up/down regulations of pathways (*P* < 0.05) in slow growing (SG) chickens. In addition, it shows the gene that harbours a genetic mutation. CS: Citrate synthase; ENO1: Enolase 1; GLUT2, 4: glucose transporter 2, 4; PKM: Pyruvate Kinase; SDHA, B: Succinate dehydrogenase complex flavoprotein subunit A, B; SLC2A4RG: Solute Carrier 2 A4 Regulator; YWHAE, Q, Z: Tyrosine 3-monooxygenase/ tryptophan 5-monooxygenase activation protein epsilon, theta, zeta

The differentially abundant proteins significantly (*P* < 0.05) enriched the metabolic pathways of ‘Translocation of GLUT4 to membrane’, ‘The Tricarboxylic Acid Cycle’, ‘Glycolysis’, ‘Gluconeogenesis’, ‘Biosynthesis of Amino Acid’, and ‘Arginine and Proline Metabolism’ (Table 1). Protein abundance for GLUT2 (Glucose transporter 2), YWHAE (Tyrosine 3-monooxygenase/tryptophan 5-monooxygenase activation protein epsilon), YWHAQ (Tyrosine 3-monooxygenase/tryptophan 5-monooxygenase activation protein theta), YWHAZ (zeta), ENO1 (Enolase 1), PKM (Pyruvate kinase), and CS (Citrate synthase) were significantly downregulated (*P* < 0.05) in SG chickens, whereas that of SDHA (Succinate dehydrogenase complex subunit A), SDHB (Succinate dehydrogenase complex subunit A), GOT1 (Glutamic-oxaloacetic transaminase 1), CK (Creatine Kinase), AGMAT (Agmatinase), and TPI1 (Triosephosphate isomerase 1) were upregulated (*P* < 0.05) (Table 2, Fig. 5, Fig. S1 in Additional file 1).

The genomic analysis identified over 900,000 variants on average in each individual (*n* = 5) with accuracy of 99.9% (Phred score ≥ 30). From those variants, 186 SNPs were on the genes of interest with deleterious effect on protein products based on Variant Effector Predictor analysis (SIFT score < .05). Of these, 15 SNP variants were affecting amino acid sensors/transporters and 5 variants affected glucose sensors/transporters (Table 3). One deleterious point mutation on the SLC2A4RG gene (20:9813052; A➔T) showed a significantly higher (Fisher’s Exact Test value = 0.007) allelic frequency in slow- compared to FG chickens (Table 3).

**Table 3:** Single nucleotide polymorphisms (SNP) with deleterious effect^a^. The SNPs identified in amino acid and glucose sensor/transporter genes in slow (SG) compared to fast (FG) growing chickens

Ligand	Transporter/Sensor	Aliases	Deleterious SNP (Chromosome: Position)	Reference Allele	Mutated Allele	SIFT score	Amino Acid Change	n (Slow Growing Chickens)	n (Fast Growing Chickens)	***P*** value (Fisher’s Exact Test)
Amino Acid	LPAR5	GPR92	1:77028001	T	C	0.00	N/D	5	3	0.44
			1:77028838	C	A	0.04	A/S	1	0	1
	SLC38A1	SNAT1	1:30954991	C	T	0.02	G/S	1	0	1
	SLC38A2	SNAT2	1:30989563	A	C	0.01	I/S	0	2	0.44
			1:30989536	C	T	0.00	G/E	0	1	1
	SLC7A11	–	4:29145636	G	A	0.00	P/L	0	1	1
	SLC7A5	LAT1	11:18195549	A	G	0.03	L/S	0	2	0.44
	SLC9A3R2	–	14:6272328	C	A	0.00	G/V	3	1	0.52
	T1R1	TAS1R1	21:564781	C	T	0.01	G/S	0	1	1
	T1R3	TAS1R3	21:2330035	G	A	0.03	H/Y	1	0	1
			21:2330503	G	C	0.00	A/G	1	0	1
			21:2328338	T	G	0.00	H/P	0	1	1
	Gust	GNAT3	1:11285686	T	G	0.00	H/P	1	0	1
	GRM7	mGluR7	12:19071629	C	A	0.00	C/F	1	0	1
			12:19098074	A	G	0.00	S/P	1	0	1
Glucose	SLC5A10	SGLT5	14:5173852	C	T	0.01	S/L	1	0	1
	SLC2A4RG^b^	GEF	20:9813052	A	T	0.00	V/E	5	0	0.007
	YWHAQ	HS1	3:96684296	A	T	0.00	L/Q	3	2	1
	OGDH	AKGDH	22:4630844	G	A	0.03	R/C	1	4	0.20
			22:4641871	C	T	0.00	D/H	4	3	1

Abbreviations: *GEF* GLUT4 enhancer factor, *GLUT4* sodium/glucose cotransporter 4, *GPR92* G protein receptor 92, *GRM7* Glutamate Metabotropic Receptor 7, *Gust* α-gustducin, *HS1* Herpes Simplex 1, *LPAR5* lysophosphatidic acid receptor 5, *OGDH* Oxoglutarate Dehydrogenase, *SGLT5* sodium/glucose cotransporter 5, *SLC38A1, 2* solute carrier family 38 member 1, 2, *SLC7A11, 5* solute carrier family 7 member 11, 5, *SLC5A10* solute carrier family 5 member 10, *SLC2A4RG* solute carrier family 2 regulator, *T1R1/3* taste receptor family 1 type 1/3, *SNAT1,2* Sodium-coupled neutral amino acid transporter 1, 2, *YWHAQ* tyrosine 3-monooxygenase/tryptophan 5-monooxygenase activation protein theta, *A* Alanine, *C* Cysteine, *D* Aspartic acid, *E* Glutamic Acid, *F* Phenylalanine, *G* Glycine, *H* Histidine, *I* Isoleucine, *L* Leucine, *N* Asparagine, *P* Proline, *Q* Glutamine, *R* Arginine, *S* Serine, *T* Threonine, *V* Valine, *W* Tryptophan, *Y* Tyrosine

^a^The table shows the 20 SNPs showing deleterious effect based on Variant Effector Predictor analysis (SIFT score < .05). Overall, the genomic analysis identified over 900,000 variants on average in each individual (*n* = 5) with an accuracy of 99.9% (Phred score ≥ 30), of which 186 SNPs were on genes of interest (Table S2 in Additional file 1)

^b^Glucose transporter SLC2A4RG presented one SNP (from A to T) with an allelic frequency significantly (*P* < 0.01) associated to growth rate (SG compared to FG) in chickens

## Discussion

When given the choice, chickens can select diets that meet their nutrient requirements [17]. We report the results of double choice preference tests used to assess selection of feeds with different amino acid content in FG compared to SG chickens. The control diet was a standard commercial feed formulated to meet or exceed the EAA requirements of the average bird of the flock. A single feed does not accommodate differences in nutrient requirements for chickens growing at different rates. FG chickens with high rates of protein deposition will be deficient while SG chickens with low rates of protein deposition will have excess of dietary amino acids. An excess of EAA or NEAA were added to the feed to identify potential dietary deficiencies. EAA cannot be synthesised by chickens, while NEAA have been defined to be synthesised from precursor compounds in chickens at a non-limiting rate.

The results confirmed a close relationship between feed intake and growth rate [18, 19], with the 10% fastest growing of a commercial flock consuming approximately 15% more feed than the 10% slowest growing. The potential amino acid deficiencies in FG chickens were compensated by an increased feed consumption compared to SG chickens. Previous reports [10, 20] described a loss of appetite and decreased feed intake in broiler chickens when excess EAA were added to a balanced diet. In our study, this response was observed only with the SG birds, reflecting the lower rate of protein deposition and a lower requirement for EAA for protein synthesis compared to FG chickens.

The opposite was true for NEAA. While rejecting the feed with excess EAA SG birds showed a strong preference for the feed with additional NEAA (see Fig. 1 A). This increased consumption of Ala, Asp and Asn showed a deliberate choice for NEAA by SG birds. These findings are consistent with the need for added NEAA in animal diets [21]. Previous work in chickens identified the relevance of dietary NEAA in sustaining or improving growth performance [22, 23]. In addition, excess of EAA or NEAA resulted in depressed feed intake in chickens showing a higher response to the essential than the non-essential [24].

EAA are not always used efficiently as a source of nitrogen [23]. Thus, an optimum ratio of EAA to NEAA in animal diets has been suggested for maximum growth and protein utilization [23, 25]. The optimum ratio in chicken diets has been identified as around 0.50 indicating twice the amount of EAA than NEAA [25, 26]. Our results showed that the optimum ratio of the three most limiting dietary amino acids in chickens (Met, Lys, Thr) with three non-essential (Ala, Asp, Asn) observed was 0.59 for FG and 0.57 for SG birds. The results suggest FG birds perceive a greater amino acid imbalance when diets contain higher concentrations of NEAA and SG birds when diets contain more EAA.

Recently, Hofman and co-workers [27] observed a decrease in growth rates in broilers fed a low crude protein diet providing adequate levels of EAA. The decrease in performance could not be compensated only by supplementing Gly (or Gly equivalent including serine) which led to the conclusion that another nutrient (possibly NEAA) was limiting the growth. In the chicken gut, neutral amino acids compete for transport. In particular, Met and Leu inhibit Ala and Gly absorption [28]. This may explain why postprandial plasma levels of Ala decreased as dietary protein levels increased above 15% in broiler chickens [29].

**Fig. 6:**
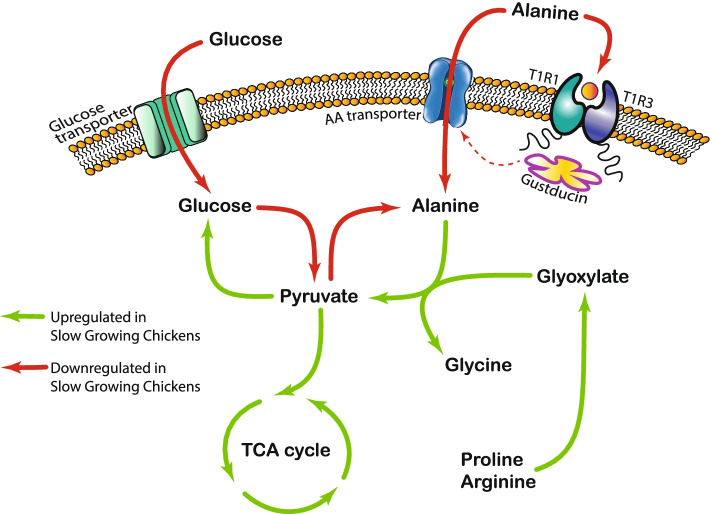
Proposed biochemical model related to alanine (Ala) and glucose metabolism in slow-growing (SG) chickens. The red arrows (with a white dot near the tip) indicate downregulation of Gustducin as a downstream signalling of umami taste receptor T1R1/T1R3 and Ala sensor in chicken, as well as downregulation of amino acid transporters (SLC1A2 and SLC38A1) suggests a downregulated sensing and transporting of Ala in SG chickens. Additionally, shortage of pyruvate due to lower glucose transportation and metabolism results in lower biosynthesis of alanine. Catabolism of Ala is upregulated due to increased pyruvate and glyoxylate metabolism feeding TCA cycle and gluconeogenesis. Therefore, SG chickens compensate the lack of glucose-derived pyruvate with Ala-derived pyruvate used as source of energy, which contribute to SG birds developing a specific appetite for Ala

The proventriculus is the glandular stomach in birds, which was selected as a proxy for the chicken gastrointestinal tract given that thanks to reverse peristalsis this organ plays a key role in feed digestion, nutrient sensing, and feed intake regulation. These mechanisms involve the secretion of hunger/satiety hormones such as ghrelin, gastrin, and cholecystokinin [30, 31]. For example, the proventriculus mediates gastrointestinal functions, including pancreatic secretions [32]. The analysis of proventricular tissues comparing SG to FG birds, showed robust differences in the catabolism of NEAA, particularly for Ala which would imply a decrease on the availability of these amino acids for protein synthesis in extra-intestinal organs such as skeletal muscle [33]. The higher catabolic rate of Ala in SG compared to FG chickens was due to a seemingly orchestrated upregulation of four metabolic pathways: the ‘glyoxylate metabolism and glycine degradation’, ‘pyruvate metabolism’, ‘gluconeogenesis’, and ‘the tricarboxylic acid cycle’ (Fig. 6).

Dietary Arg and Pro are partially metabolised in the intestine [34]. We observed an upregulation of the ‘arginine and proline metabolism’ in SG birds resulting in the production of glyoxylate via hydroxyproline further increasing the need for Ala [35–37]. Shortage of Ala can lead to accumulation of glyoxylate, which converts to oxalate [38]. Oxalate is an anti-nutritional compound present in cereal grains such as wheat and barley and in pulses such as soybean. Ala, as an amino group donor, has a key role in detoxification of oxalate and glyoxylate via Alanine-glyoxylate transaminase (AGT) [38–40].

Higher consumption of aspartic acid in SG chickens could be explained by the upregulation of the ‘arginine and proline metabolism’ and the ‘gluconeogenesis’ metabolic pathways (Fig. 6, Fig. S1 in Additional file 1). Aspartate-arginosuccinate shunt is the pathway that connects urea cycle to tricarboxylic acid (TCA) cycle [41, 42]. Upregulation of the key enzyme GOT1 in SG birds suggests the potential use of Asp, along with Ala as a source of energy and a key intermediate for the urea cycle.

In chickens, Ala is the primary transporter of nitrogen and hydrocarbon molecules between liver and skeletal muscles [43, 44]. A deficiency of Ala uptake in the gut lumen can reduce the availability of the amino group and hydrocarbon skeleton for amino acid and protein synthesis in extra-intestinal organs. SG compared to FG chickens had lower gene expression levels of the amino acid transporters SLC38A1 and SLC1A2. SLC38A1 belongs to the SLC38A family, which have been related to Ala and Gln transport in mammals [45]. In addition, the alpha subunit of Gustducin, the heterotrimeric G-protein coupled to dimeric amino acid sensors T1R1/T1R3, was also downregulated in the SG birds. In chickens, the main ligand to the dimeric T1R1/T1R3 amino acid sensor known to date is Ala [46]. These observations strongly indicate that SG chickens are less efficient in sensing and transporting Ala, making it a limiting intermediate metabolite for protein accretion and growth.

This study also highlighted significant differences in glucose transport and metabolism between SG and FG chickens. Glucose uptake in slow growers seemed to be impaired as a result of the downregulation of the glucose transporters GLUT1, GLUT2, and GLUT10 (coding genes: SLC2A1, SLC2A2, and SLC2A10, respectively). Furthermore, a deleterious mutation was identified in the SLC2A4RG gene (protein name: GLUT4RG), which is the transcription factor for the glucose transporter GLUT4, coding gene SLC2A4 [47, 48]. The insulin-stimulated translocation to the cytosolic membrane of GLUT4 may also be impaired in SG chickens. This impairment may be explained by the downregulation of the main proteins responsible for the translocation, YWHAE, YWHAQ, and YWHAZ, in SG compared to FG chickens [49–51]. Taken together, the data strongly suggests that slow growers have a limited capacity for glucose sensing and uptake relative to fast growers. The point mutation on SLC2A4RG warrants more investigation. It would be interesting to study if this mutation is the main cause of metabolic differences between SG and FG which has not been addressed in this study. Alternatively, for future studies, the genotyping a bigger chicken population (related to this SNP) and/or the application of gene editing technologies may help understand the function of SLC2A4RG in the chicken.

The downregulation of ‘glycolysis’, the metabolic pathway involved in the conversion of glucose to pyruvate, together with the upregulation of ‘pyruvate metabolism’ (converting pyruvate to Acetyl-CoA), ‘gluconeogenesis’ (synthesising glucose from NEAA and oxaloacetate) and ‘the tricarboxylic acid cycle’ would converge in causing a shortage of pyruvate in SG birds. An upregulated TCA cycle such as in SG individuals consumes more pyruvate, whereas the results showed that production of pyruvate is less due to downregulated glycolysis. The scarcity of pyruvate in the cell would then have implications relevant to the cellular availability and production of Ala. Ala inhibits pyruvate kinase to slow glycolysis and induce gluconeogenesis with increased production of glucose from Ala [52, 53]. First, Ala will be catabolized to pyruvate to feed the TCA cycle for energy production [54], and second, low pyruvate concentration will reduce the biosynthesis of Ala [52]. Importantly, the gut uses primarily NEAA rather than glucose or EAA to produce ATP [33, 55]. Thus, a lower availability of NEAA, particularly Ala, in the intestinal cells would impair nutrient transport and absorption in the gut. Our current results provide evidence of the latter occurring in SG chickens.

A pathway illustrating the key associations between growth rate and Ala and glucose metabolism in chickens is proposed in Fig. 6. The orchestrated variations in metabolic pathways observed in SG chickens seem to lead to cytosolic glucose, pyruvate and Ala scarcity. Cereal-based diets provide a high starch content (> 50%) indicating that dietary glucose is unlikely to be a limiting nutrient in this study. The data presented is consistent with previous reports showing that feed intake and growth can be improved with dietary NEAA supplementation in chickens [7]. In summary, the downregulation of Gustductin through action of the umami taste receptor T1R1/T1R3 and Ala sensor combined with downregulation of amino acid transporter genes SLC1A2 and SLC38A1 results in reduced sensing and reduced transport of Ala in SG birds. Decreased glucose transport into the cell and reduced glycolysis would cause low cytosolic abundance of pyruvate while reducing Ala biosynthesis. In addition, Ala catabolism was enhanced due to an increased demand for TCA cycle intermediates for gluconeogenesis. SG chickens seem to upregulate the synthesis of pyruvate from Ala to feed the TCA cycle while compensating for the lack of glucose-derived pyruvate. These metabolic changes result in a scarcity of cytosolic Ala, which is reflected in a specific appetite for Ala observed in the feeding choice tests. The results presented provide strong evidence that chickens with lower growing rates may require additional dietary NEAA, particularly Ala.

## Conclusion

SG compared to FG chickens select diets containing less EAA (Lys, Met, Thr) and more NEAA (Ala, Asp, Asn) in double choice preference tests. The difference in preference reflects differences in the rate of protein synthesis and dietary amino acid requirements. The differential expression of genes and different abundance of proteins point to a robust impairment in the sensing and transporting of Ala and glucose in SG compared to FG chickens. In addition, Ala catabolism is upregulated to compensate the deficiency of pyruvate required for the TCA cycle in a gluconeogenic status. The subsequent depletion of cytosolic Ala would explain the preference for the NEAA in SG chickens. Dietary supplements of NEAA have the potential to decrease variability in feed intake and growth in young animals.

## Methods

### Behavioural studies

#### Double-choice tests

Body weight at 3 weeks of age was used to select FG and SG chickens from a commercial flock of Ross 308 birds (Darwalla Group, Qld, Australia). The weight of 100, randomly selected, birds was used to identify those birds weighing < 800 g as SG and those weighing > 1000 g as FG, with each category representing approximately 10% of the flock. Birds were randomly selected from the flock until there were 36 in each of the FG and SG categories. The selected birds were transferred to individual cages (45 × 35 × 35 cm) equipped with one nipple drinker and two identical feeders (16 × 10 × 7 cm) placed side-by-side. The 3-week-old chickens were kept for another 3 weeks for the experimental phase. They were offered a commercial standard grower feed for the first week of adaptation period, and a commercial standard finisher feed for the following two weeks as part of the double-choice (DC) testing period. The ingredient and nutrient composition of the two standard commercial feeds (Darwalla Group, Mt Cotton, Queensland, Australia) feeds can be found in Table S1 in Additional file 1.

The DC tests were conducted to study feed preference based on EAA or NEAA supplementation by offering ad libitum access to the two feeders one containing the control finisher feed and the other the experimental (supplemented) feed. The preparation of the experimental feeds consisted in spraying the base commercial finisher feed with water (control), or water containing a mix of three EAA (methionine, Met; lysine, Lys; and threonine, Thr) or three NEAA (alanine, Ala; aspartic acid, Asp; asparagine, Asn). The content of amino acids of interest in the control and the EAA or NEAA supplemented diets is presented in Table 4. The added EAA or NEAA were 73% above the amounts present in the control feed because this level of excess was found as the maximum inclusion of EAA not having a significant (*P* > 0.05) effect on feed intake or growth compared to optimum levels in broiler chickens [56, 57]. The amount of each feed consumed was measured every 24 hours. After every measurement, feeders’ position was switched to avoid errors due to side preference. Preference (%) for a feed was determined as a percentage of the experimental feed consumed divided by the total amount of both feeds consumed.

**Table 4:** Description of the treatments. Content of essential (Met, Lys, Thr) and non-essential (Ala, Asp and Asn) amino acids in the control^a^ and supplemented feeds^b^ offered in double-choice (DC) tests (T1, T2 or T3)^c^ performed in slow (SG)- and fast-growing (FG) chickens

Amino Acid	Type	T1: Control feed (%)^**a**^	T2: EAA Supplemented feed (%)^c^	T3: NEAA Supplemented feed (%)^c^
Methionine (Met)	EAA	0.428	0.740	–
Lysine (Lys)	EAA	1.626	2.813	–
Threonine (Thr)	EAA	1.161	2.009	–
Alanine (Ala)	NEAA	1.427	–	2.468
Aspartic acid (Asp)	NEAA	2.735	–	4.732
Asparagine (Asn)	NEAA	1.368	–	2.366

Acronyms: *EAA* essential amino acids, *NEAA* non-essential amino acids

^a^The control feed was a standard commercial broiler feed (Darwalla Group, Esk, QLD, Australia). See Table S1 in Additional file 1

^b^The EAA or NEAA supplemented feeds consisted of the control diet supplemented with a mix of Met, Lys and Thr (EAA) or Ala, Asp and Asn (NEAA), respectively. All amino acids were supplemented to reach 73% excess of the control diet, the maximum level with no significant impact on feed intake (Baker and Han, 1994; Mack et al., 1999)

^c^The double-choice (DC) tests T1, T2 and T3 consisted of a control DC offering two identical control feeds (T1) or a DC between the control feed and either the EAA (T2), or the NEAA (T3) supplemented feeds

### Omics studies

#### Animal and tissue sampling

The 48 lightest and 48 heaviest birds at 3 weeks of age from a batch of 580 male (feather-sexed) Ross 308 chickens (Darwalla Group, Qld, Australia) were selected as SG or FG chickens, respectively. Chickens showing any sign of pathology were not included. The birds were transferred to individual cages similar to those used for the DC experiments and offered a commercial diet until 6 weeks old. Individual cages allowed for individual feed intake and body weight recording. The five lightest and five heaviest birds were selected for the metabolic studies and euthanised using cervical dislocation. Cervical dislocation is a common method of euthanasia used in poultry research experiments where the bird’s neck is quickly pulled by hand leading to dislocation of the first cervical vertebrae with severing of the spinal cord and carotid arteries. This method of euthanasia does not involve the use of any chemical or equipment. Every bird was confirmed to be male using post-mortem and DNA sexing (MDS Australia Pty Ltd., Queanbeyan, Australia). A sample (400-500 mg) of the proventriculus taken from each bird was placed in 1 ml RNA-later solution (ThermoFisher Scientific, Waltham, USA) and stored at − 80 °C.

#### RNA sample preparation and sequencing

RNA was extracted from 30 to 40 mg of tissue using the Maxwell® 16 LEV Simply RNA Purification Kit manufactured by Promega Corporation, Madison, USA. The quality and quantity of the RNA samples were examined using a NanoDrop™ spectrophotometer (ThermoFisher Scientific, Waltham, USA) and Agilent 2100 Bioanalyzer (Agilent Technologies, Santa Clara, USA). RNA samples with an Integrity Number ≥ 8, a 260/280 ratio between 2 and 2.1, and a 260/230 ratio between 2 and 2.2 were used for sequencing. An RNA sample of 500-4000 ng from each SG and FG bird was sequenced using high throughput sequencing (Queensland Brain Institute, University of Queensland, Australia). A TruSeq Stranded mRNA Library Prep Kit was used for library construction. Sequencing and coverage were based on 2 × 125 bp Paired-End Dual indexed reads and v4 illumina SBS chemistry (Illumina, San Diego, USA), with HiSeq 2000 used to produce an average of ~ 27 million sequence reads per sample.

The RNA-seq data was validated using RT-qPCR analysis. The detailed method is provided in the Additional file 1.

#### Protein sample preparation

Approximately 20 mg of tissue was used for protein extraction and Mass Spectrometry. The tissue was placed in ~ 300 μl of lysis buffer (6 M Guanidine Chloride, 50 mM Tris pH 8, and 10 mM DTT) and sonicated for 10 seconds before being vortexed at 30 °C for 1 hour. Acrylamide (25 mM) was added and incubated for 1 h before adding 5 mM DTT. Protein was precipitated by adding 4 volumes of 1:1 methanol acetone and stored overnight at − 20 °C before centrifuged to remove the solvent, and the pellet suspended in 0.1% SDS (Sodium dodecyl sulfate). The protein concentration of each sample was measured using NanoDrop (ThermoFisher Scientific, Waltham, USA). Samples were digested with trypsin overnight at 37 °C using an Amicon column and ammonium bicarbonate (50 mM). Samples were desalted by C-18 Zip-tip (adapted from Millipore procedure) and analyzed by liquid chromatography electrospray ionization tandem mass spectrometry (LC-ESI-MS/MS).

#### Mass spectrometry and data analysis

LC-MS/MS analysis was performed using a Prominence nanoLC system (Shimadzu, Kyoto, Japan) and TripleToF 5600 mass spectrometer with a Nanospray III interface (SCIEX, Toronto, Canada). A 70 min Liquid chromatography (LC) gradient was used to separate the peptides. The database of Protein Pilot (Uniprot, www.uniprot.org) was used to identify peptides and proteins. Sequential window acquisition of all theoretical mass spectra (SWATH) was performed on all samples. An information-dependent acquisition library (IDA) was prepared from one randomly chosen slow grower sample and one randomly chosen fast grower sample for each tissue.

#### Transcriptomic and genomic analysis

For transcriptomic analysis, sequenced reads were first put through a quality check (QC) using FastQC version 0.72 (Babraham Bioinformatics, Babraham Institute, Cambridge, UK) in Galaxy Australia platform with default parameters [58]. Then, the reads were mapped to Galgal.5 reference assembly using aligners HISAT2 and Salmon with default settings [59, 60]. InteractiVenn, JMP, IGV, and MeV were used to visualize and perform cluster and Principal Component Analyses (PCA) [61, 62].

As for genomics, Galaxy Australia was used to perform the analyses [58]. RNA sequences were mapped on the reference genome (Galgal.5) using Bowtie2, version 2.3.4.1 [63]. Genomic variants between the 5 SG and 5 FG chickens were identified using FreeBayes, which is a Bayesian genetic variant detector designed to find small polymorphisms, specifically SNPs (single-nucleotide polymorphisms), indels (insertions and deletions), MNPs (multi-nucleotide polymorphisms), and complex events (composite insertion and substitution events). The list of candidate genes used for the study of genomic variants is presented in Table S2 in Additional file 1. The FreeBayes output was first filtered for Phred score ≥ 30 to attain accuracy of 99.9%. Phred score is an index of variant quality score indicating the confidence level in detecting a particular variant accurately [64]. Next, the FreeBayes output was filtered for SNPs considered of interest in relations to metabolic functions of chickens growing at different rates. These SNPs were further filtered for a SIFT (Sorting Intolerant From Tolerant) score of < 0.05, which predicts the extent to which the polymorphism effects gene function, with 0.0 being deleterious and 1.0 being tolerable in Variant Effect Predictor (VEP) analysis.

#### Functional enrichment analysis

Differentially expressed genes (DEG) and differentially abundant proteins (DAP) between the FG and SG groups were used as input for metabolic pathway enrichment analyses. Enrichment analyses were performed in DAVID 6.8 [65]. Enriched metabolic pathways and terms in different databases including Gene Ontology, GO [66], Kyoto Encyclopedia of Genes and Genomes, KEGG [67], and REACTOME [68] were used to attain insight into the function of DEGs and DAPs.

### Statistical analyses

#### Preference tests

A 2 × 3 factorial design was used including two growth rates for SG or FG chickens and three preference comparisons of feed vs feed plus amino acids (Table 4). Preference for each feed in a DC test was compared to 50%, which is the neutral value, using t-student test. Main effects and interactions were analysed using the general linear model (GLM) procedure of SAS9.4 (SAS Institute, Cary, North Carolina, United States), with *P* < 0.05 as the level of significance in Tukey test. The statistical model to compare the preference and individual feed intakes was:

$${\mathrm{y}}_{\mathrm{i}\mathrm{j}\mathrm{k}}=\mu +{\mathrm{A}}_{\mathrm{i}}+{\mathrm{B}}_{\mathrm{j}}+{\left(\mathrm{AB}\right)}_{\mathrm{i}\mathrm{j}}+{\varepsilon}_{\mathrm{i}\mathrm{j}\mathrm{k}}\kern0.5em \mathrm{i}=1-2;\mathrm{j}=1-3;\mathrm{k}=1-6$$

y_ijk_ = observation k in level i of group of chicken and level j of treatment; μ = the overall mean; A_i_ = the effect of level i of group of chicken (slow vs fast grower); B_j_ = the effect of level j of treatment; (AB)_ij_ = the effect of the interaction of level i of chicken group with level j of treatment; ε_ijk_ = random error; i = Number of levels of chicken group; j = Levels of treatment; k = biological replicates.

Total feed intake was used as a covariate for comparisons between treatments and controls and for amino acids consumed. The covariate allowed comparison between SG and FG chickens while adjusting the treatment effect for the variability in the feed intake.

Randomized design with a covariate was also run in GLM procedure of SAS9.4 according to the statistical model below.

$${\displaystyle \begin{array}{cc}{\mathrm{y}}_{\mathrm{i}\mathrm{jk}}={\beta}_0+{\beta}_1{\mathrm{x}}_{\mathrm{i}\mathrm{jk}}+{\tau}_{\mathrm{i}}+{\mathrm{g}}_{\mathrm{j}}+{\varepsilon}_{\mathrm{i}\mathrm{jk}}& \mathrm{i}=1-3;\mathrm{j}=1-2;\mathrm{k}=1-6\end{array}}$$

y_ijk_ = observation k in treatment i group j; β_0_ = the intercept; β_1_ = the regression coefficient; x_ijk_ = a continuous independent variable of total feed intake (covariate); τ_i_ = the fixed effect of treatment (T1–3); g_j_ = the fix effect of group (slow vs fast grower); ε_ijk_ = random error.

#### Metabolic studies

For transcriptomics, read count and differential expression analysis were performed using Limma [69]. Limma provides a linear model capable of multiple RNA comparisons in the context of multifactor design experiments to identify differentially expressed genes (DEG). The basic statistics used for DEG identification was t-statistics moderated across genes using a Bayesian model. This will increase the reliability of the results. Significance threshold was set as a *P*-value of 0.05 or less.

As for proteomics, sequential window acquisition of all theoretical mass spectra (SWATH)-MS relative quantitative data were analysed using PeakView v2.1 (SCIEX) [70]. To identify differentially abundant proteins (DAP), statistical analyses were performed using MSstats in R as previously described [71, 72]. Values less than 0.05, adjusted for multiple testing, were considered as significant *P*-values.

Pathway enrichment analyses were performed on both DEG and DAP lists. Pathways that were statistically (*P* < 0.05) over-represented in the DEG and DEP lists were defined as significantly enriched. This was done through Fuzzy clustering algorithms and Fisher’s exact test built in DAVID bioinformatics platform [65].

Regarding genomic variants, allelic frequencies were compared between SG and FG chickens using Fisher’s exact test in SAS 9.2 (SAS Institute, Cary, North Carolina, United States). P-values of 0.05 or less were considered as significant for all allelic frequency comparisons.

## Supplementary Information

**Additional file 1.**

## Data Availability

The raw datasets generated and analysed during the current study are available in the National Centre for Biotechnology Information (NCBI), Sequence Read Archive (SRA) under accession number PRJNA771098 (https://submit.ncbi.nlm.nih.gov/).

## Abbreviations

AGMAT: Agmatinase
AGT: Alanine-glyoxylate transaminase
Ala: Alanine
Asn: Asparagine
Asp: Aspartic Acid
ATP: Adenosine triphosphate
CCK: Cholecystokinin
CEAA: Conditionally essential amino acid
CK: Creatine Kinase
CS: Citrate synthase
Cys: Cysteine
DAP: Differentially abundant proteins
DEG: Differentially expressed genes
DC: Double choice test
EAA: Essential amino acid
ENO1: Enolase 1
FAO: Food and Agriculture Organization
GLUT1: Glucose transporter 1
GLUT2: Glucose transporter 2
GLUT4: Glucose transporter 4
GLUT10: Glucose transporter 10
GLUT11: Glucose transporter 11
GLUT13: Glucose transporter 13
GLUT4RG: Glucose transporter 4 Regulator
GOT1: Glutamic-oxaloacetic transaminase 1
Gust: Gustducin
His: Histidine
indels: Insertions and deletions
LC-ESI-MS/MS: Liquid chromatography electrospray ionization tandem mass spectrometry
LIAS: Lipoic acid synthetase
Lys: Lysine
Met: Methionine
MNP: Multi-nucleotide polymorphisms
NEAA: Non-essential amino acid
OGDH: Oxoglutarate (α-ketoglutarate) dehydrogenase
PDHB: Pyruvate dehydrogenase beta
PDHX: Pyruvate dehydrogenase complex component
PKM: Pyruvate kinase
SDHA: Succinate dehydrogenase complex subunit A
SDHB: Succinate dehydrogenase complex subunit B
Ser: Serine
SLC: Solute Carrier gene family
SLC1A2: Solute carrier 1 type A2
SLC2A1: Solute carrier 2 type A1
SLC2A4RG: Solute carrier 2 type A4 Regulator
SLC2A10: Solute carrier 2 type A10
SLC2A11: Solute carrier 2 type A11
SLC38A1: Solute carrier 38 type A1
SNP: Single-nucleotide polymorphisms
SWATH: Sequential window acquisition of all theoretical mass spectra
T1R1/T1R3: Umami taste receptor
TCA: Tricarboxylic acid
Thr: Threonine
TPI1: Triosephosphate isomerase 1
VEP: Variant Effect Predictor
YWHAE: Tyrosine 3-monooxygenase/ tryptophan 5-monooxygenase activation protein epsilon
YWHAQ: Tyrosine 3-monooxygenase/ tryptophan 5-monooxygenase activation protein theta
YWHAZ: Tyrosine 3-monooxygenase/ tryptophan 5-monooxygenase activation protein zeta
