# Supplementary material for: Alanine-specific appetite in slow growing chickens is associated with impaired glucose transport and TCA cycle
Source: BMC Genomics. 2022 May 23;23:393. doi: 10.1186/s12864-022-08625-2 (PMC9128104; doi:10.1186/s12864-022-08625-2)
Supplement: Supplementary file 1 — Additional file 1. [file 12864_2022_8625_MOESM1_ESM.docx]

**Alanine-specific appetite in slow growing chickens is associated with impaired glucose transport and TCA cycle**

Shahram Niknafs^1^, Marina R S Fortes^2^, Sungbo Cho^1^, John L Black^3^, Eugeni Roura^1^

^1^ Centre for Nutrition and Food Sciences, Queensland Alliance for Agriculture and Food Innovation, The University of Queensland, St. Lucia, QLD 4072, Australia; ^2^ School of Chemistry and Molecular Bioscience, The University of Queensland, St Lucia, Qld 4072, Australia; ^3^ John L Black Consulting, Warrimoo, NSW 2774, Australia

[s.niknafs@uq.edu.au](mailto:s.niknafs@uq.edu.au)

[m.fortes@uq.edu.au](mailto:m.fortes@uq.edu.au)

[blue0555@hotmail.com](mailto:blue0555@hotmail.com)

[jblack@pnc.com.au](mailto:jblack@pnc.com.au)

[e.roura@uq.edu.au](mailto:e.roura@uq.edu.au)

Corresponding Author:

Professor Eugeni Roura, Room S435, Hartley Teakle [#83], The University of Queensland, St Lucia, Brisbane 4072, Australia. Tel: +61 7 3365 2526, E: [e.roura@uq.edu.au](mailto:e.roura@uq.edu.au)

**Supplementary Materials and Methods:**

**RT-qPCR validation**

RNA samples used for RNAseq analyses and the same extraction method explained in the main text of the manuscript were used for RT-qPCR validation of the RNAseq. For cDNA synthesis, QantiTect Reverse Transcription Kit (QIAGEN, Hilden, Germany) was used according to the manufacturer’s instructions. Briefly, 1µg of RNA was mixed with 2µl of gDNA Wipeout buffer and reached to 14µl volume by adding RNase-free water. The mixture was incubated at 42ºC for 2min to eliminate the genomic DNA in the samples. Reverse Transcriptase (RT) at 1µl of, 4µl of RT buffer, and 1µl primer mixed were added to the samples to reach final volume of 20µl. Samples were incubated for 30min/42ºC followed by 3min/95ºC. In order to check for any genomic DNA contamination in the samples, we prepared RT negative reactions for each sample. Both RT positive and negative were tested using conventional PCR following the standard protocol for Taq PCR Kit from KAPABIOSYSTEMS (Wilmington, USA). A cDNA pool was prepared and specificity of all the primers (Table below) were checked before RT-qPCR.

Several nutrient sensing genes and amino acid transporters known to be expressed in the gastrointestinal tract of chickens were used as reporter genes to validate the RNAseq analysis. The selected genes were TAS1R1/TAS1R3; GPR92 and 139: G protein coupled receptor 92 and 139; CaSR: Ca sensing receptor, Gust: α-gustducin, B-ACT: Beta actin, and GAPDH: Glyceraldehyde 3-phosphate dehydrogenase. These genes were selected because they are known to be involved in sensing and transporting amino acids in the gastrointestinal tract (GIT) across different species. All primers were designed using the Pick Primers option on the NCBI database according to published cDNA sequences (Table below). Specificity of the primers were confirmed by conventional PCR and agarose gel to ensure that the correct DNA fragment is amplified. The annealing temperature was set at 60±2 ºC, and the amplicon length was defined to be 100-300 bp (without spanning the exon-exon junction). Total volume of 10 µl (1 µl cDNA, 1 µl primers, 3 µl water, and 5 µl SYBER green) were used for real-time PCR reactions. Samples were run in triplicate, and two reference genes *GAPDH* and *β-ACT* were used to normalize the data. Each tissue was run on a different plate, and in order to be able to compare across the plates (tissues), positive controls for each gene using pooled cDNA with three technical replicates was considered in all the plates. Plates were run on Applied Biosystems RT-qPCR instrument (Applied Biosystems, Foster City, California, United States) with the following program: 50ºC for 2 min, 95ºC for 10 min, and 40 cycles of 95ºC for 15 s and 60ºC for 1min. The melt curve stage was done under the following conditions: 95ºC for 15 s, 60ºC for 1 min with increment rate of 0.05ºC/s to reach 95ºC for 15 s.

RT-qPCR data was analysed using the Pfaffl method (see Equation below) (Pfaffl, 2001). To do so, the PCR efficiency of each primer was calculated using delta Rn information which was calculated using the LinRegPCR software (Ruijter et al., 2009). The Pfaffl method calculates the expression ratio by normalizing the Ct values (threshold cycle number) using reference genes and plate control simultaneously, so that all experimental conditions, target genes and tissues can be compared.

$$Normalized Expression Ratio= \frac{{(E_{\mathrm{target}})}^{{{\Delta CP}_{\mathrm{target}}}^{(control-sample)}}}{{(E_{\mathrm{ref}})}^{{{\Delta CP}_{\mathrm{ref}}}^{(control-sample)}}}$$

*E_target_* and *E_ref_* = efficiency of target and reference gene respectively, *∆CP_target_* (control - sample) = difference between average Ct value of positive control and sample with target gene, *∆CP_ref_* (control - sample) = difference between average Ct value of positive control and sample with reference gene

After calculating normalized expression ratios, the GLM procedure of SAS 9.4 (SAS Institute, Cary, North Carolina, United States) was used to perform the statistical analysis for gene expression level comparison between fast- and slow-growing chickens in duodenum and proventriculus. A 2x2 factorial design was used in which two levels of group effect (slow- vs fast-growing) and two levels of tissue effect (duodenum and proventriculus) were considered. The following model was used to run the statistical analysis:

***y_ijk_* = *μ* + *G_i_* + *T_j_* +(*GT*)*_ij_* + *ε_ijk_ i* = 1-2; *j* = 1-2; *k* = 1-6**

***y_ijk_*** = observation *k* in level *i* of group of chicken and type *j* of tissue

***μ*** = the overall mean

***G_i_*** = the effect of level *i* of group of chicken (slow vs fast grower)

***T_j_*** = the effect of type *j* of tissue

**(*GT*)*_ij_*** = the effect of the interaction of level *i* of chicken group with type *j* of tissue

***ε_ijk_*** = random error

***i =*** Number of levels of chicken group; ***j*** = Type of tissue; ***k*** = biological replicates

Target genes and primer sequences used in the experiment to measure relative mRNA abundance of AA sensors in the GIT of slow- vs fast-growing chickens (Niknafs et al, 2018).

| **Gene** | **Primer (5’-3’)** | **Amplicon length (bp)** | **Accession No** |
| --- | --- | --- | --- |
| T1R3 | F - ACAACTCCACGTCACTCCTG  R - CCATAGCTGACCTGTGGAATCA | 253 | XM_015297004.1 |
| T1R1 | F - CTATGGTAGGGATGGGCTCAAC  R - CTAAAGACCAGTCCTCAGAGCC | 260 | XM_015297117.1 |
| Gustducin | F - AACCACTTCCATCGTGCTGT  R - GGCACAGGTCAGGTGACAAT | 199 | NM_001267811.1 |
| GPR92 | F - GGACAAACCTGGCACTCAGA  R - GCTAGGGGCTTTCTGTGGTT | 207 | XM_015293753.1 |
| GPR139 | F - TGCTGACATCCTCGTTCTCTT  R - GAGTGGATGGCACACAGCTA | 190 | NM_001321735.1 |
| CaSR | F - TGGCTTCCACCTTGTTGCTTA  R - GCAGCAGTGTTCCAGGTAAAC | 214 | XM_416491.5 |
| GAPDH | F - GTAGTGAAGGCTGCTGCTGA  R - TCCTTGGATGCCATGTGGAC | 224 | NM_204305.1 |
| Β-ACT | F - GAGAAATTGTGCGTGACATCA  R - CCTGAACCTCTCATTGCCA | 152 | NM_205518.1 |

**Supplementary Results:**


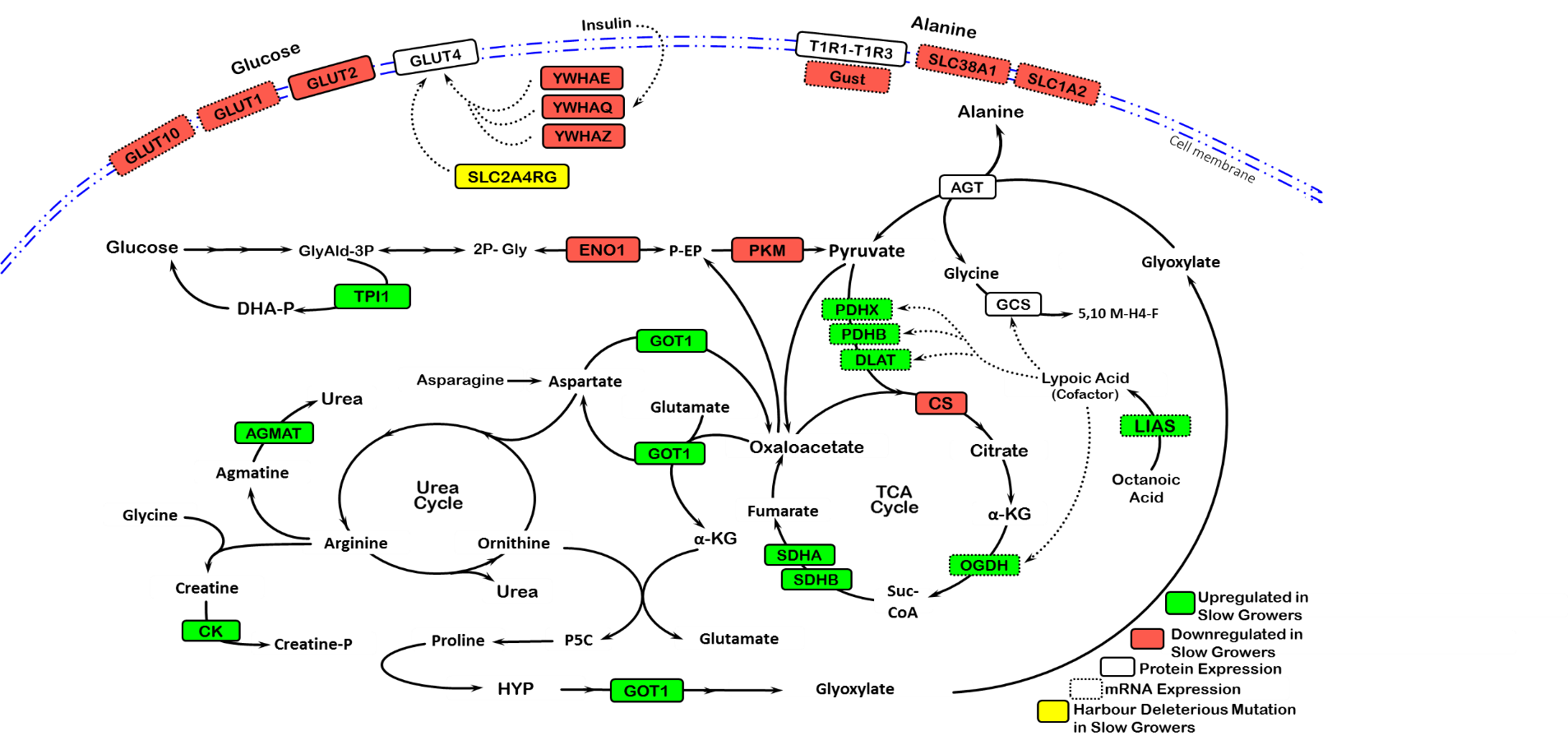


**Figure S1**. Integrated details of the enriched pathways from transcriptomic, proteomic, and genomic data representing their up/down regulations (P<0.05) in slow-growing chickens (AGMAT: Agmatinase; AGT: Alanine-glyoxylate transaminase; CK: Creatine Kinase; CS: Citrate synthase; DLAT: dihydrolipoamide S-acetyltransferase; ENO1: Enolase 1; GCS: Glycine cleavage system; GLUT1, 2, 4, 10: glucose transporter 1, 2, 4, 10; GOT1: Glutamic-oxaloacetic transaminase 1; Gust: Gustducin; LIAS: Lipoic acid synthetase; OAA: Oxaloacetate; OGDH: oxoglutarate (α-ketoglutarate) dehydrogenase; PDHB: pyruvate dehydrogenase (lipoamide) beta; PDHX: pyruvate dehydrogenase complex component; PKM: Pyruvate Kinase; PRPS2: Phosphoribosyl pyrophosphate synthetase 2; SDHA, B: Succinate dehydrogenase complex flavoprotein subunit A, B; SLC2A4RG: Solute Carrier 2 A4 Regulator; SLC38A1: Solute Carrier 38 A1; SLC1A2: Solute Carrier 1 A2; TPI1: Triosephosphate isomerase 1; T1R1-T1R3: Umami taste receptor; YWHAE, Q, Z: Tyrosine 3-monooxygenase/ tryptophan 5-monooxygenase activation protein epsilon, theta, zeta).


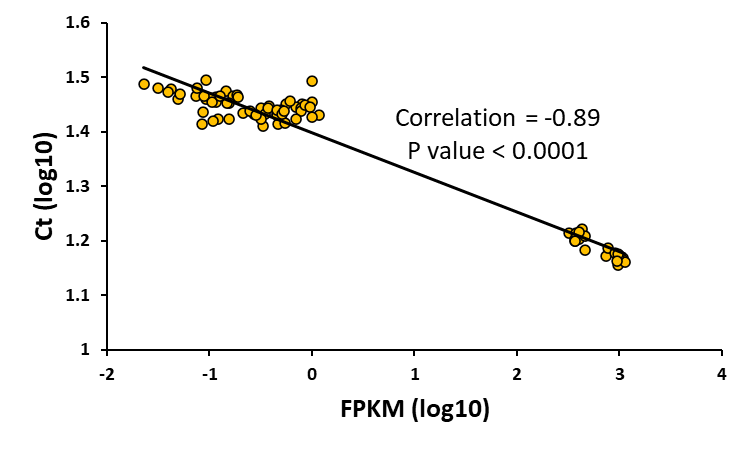


**Figure S2.** Correlation between gene expression level of 8 candidate genes using RNAseq and qPCR. Ct: CT value; FPKM: Fragments Per Kilobase of transcript per Million mapped reads. The Results showed a high correlation coefficient (-0.89; P<0.0001) between gene expression of these genes measured using qPCR (Ct value) and RNAseq (RPKM)

**Table S1.** Ingredient and nutrient composition of the control^1^ and supplemental^2^ diet used in the double-choice experiments.

|  | **Item** | **% in different diets** | |
| --- | --- | --- | --- |
|  |  | **Grower** | **Finisher** |
| **Ingredient** | Wheat | 30 | 30 |
|  | Sorghum | 32.7 | 39.5 |
|  | Soybean Meal | 19.5 | 13 |
|  | Canola Meal | 7 | 8 |
|  | Meat Meal | 4.2 | 3.63 |
|  | Blood Meal | - | - |
|  | Poultry Oil (press) | 3.64 | 3.06 |
|  | Poultry Oil (mixer) | 1.5 | 1.5 |
|  | Alimet (Met) | 0.33 | 0.25 |
|  | Lysine | 0.32 | 0.31 |
|  | Broiler starter premix | 0.33 | 0.33 |
|  | Sodium Bicarbonate | 0.14 | 0.15 |
|  | Salt | 0.075 | 0.151 |
|  | Threonine | 0.089 | 0.070 |
|  | Choline Chloride | 0.033 | 0.033 |
| **Nutrient** | Protein | 21.6 | 19.29 |
|  | Fat | 7.31 | 6.80 |
|  | Fibre | 3.27 | 3.21 |
|  | Calcium | 0.87 | 0.78 |
|  | Available phosphorus | 0.51 | 0.477 |
|  | Methionine | 0.58 | 0.489 |
|  | Methionine + Cysteine | 0.94 | 0.828 |
|  | Lysine | 1.23 | 1.059 |
|  | Tryptophan | 0.245 | 0.223 |
|  | Arginine | 1.26 | 1.07 |
|  | Threonine | 0.82 | 0.721 |
|  | Isoleucine | 0.83 | 0.736 |
|  | Valine | 0.97 | 0.866 |
|  | Leucine | 1.64 | 1.50 |
|  | Choline (mg/kg) | 1421.2 | 1298.8 |
|  | Metabolizable Energy (MJ/kg) | 13.2 | 13.3 |
|  | Phosphorus | 0.66 | 0.613 |
|  | Digestible Methionine | 0.51 | 0.43 |
|  | Digestible Met + Cys | 0.83 | 0.733 |
|  | Digestible Lysine | 1.10 | 0.94 |
|  | Sodium | 0.16 | 0.16 |
|  | Potassium | 0.757 | 0.65 |
|  | Chloride | 0.190 | 0.19 |
|  | Digestible Threonine | 0.726 | 0.63 |
|  | Digestible Tryptophan | 0.237 | 0.207 |
|  | Digestible Isoleucine | 0.764 | 0.669 |
|  | Digestible Leucine | 1.44 | 1.32 |
|  | Digestible Arginine | 1.14 | 0.96 |
|  | Digestible Valine | 0.855 | 0.759 |

^1^The control diet was a standard broiler feed supplied by the Darwalla Group (Esk, QLD, Australia).

^2^The supplemental feeds consisted of the control diet described in the table supplemented with a mix of three essential (Met, Lys and Thr) or three non-essential (Ala, Asp, and Asn) amino acids. Each amino acid was supplemented to reach 73% excess of the control diet.

**Table S2.** List of candidate genes that were used for genomic variant calling

| **Gene** | Aliases | **Entrez ID** | **Chr** | **Gene** | Aliases | **Entrez ID** | **Chr** |
| --- | --- | --- | --- | --- | --- | --- | --- |
| **CASR** | - | 418266 | 1 | **SLC2A2** | GLUT-2 | 396272 | 9 |
| **GPRC6A** | - | 428620 | 3 | **SLC2A14** | GLUT3, SLC2A3 | 396517 | 1 |
| **GPR92** | LPAR5 | 418279 | 1 | **SLC7A6** | y+ LAT-2 | 415622 | 11 |
| **GPR139** | - | 431418 | 14 | **SLC7A5** | LAT1 | 415832 | 11 |
| **CD36** | - | 417730 | 1 | **SLC9A3R2** | - | 416550 | 14 |
| **T1R1** | TAS1R1 | 428176 | 21 | **SLC2A4** | GLUT4, SLC2A4 | 417384 | 18 |
| **T1R3** | TAS1R3 | 428182 | 21 | **SLC43A2** | LAT4 | 417557 | 19 |
| **SGLT1** | SLC5A1 | 395496 | 15 | **SLC38A1** | SNAT1 | 417806 | 1 |
| **GPR120** | FFAR4; O3FAR1 | 428963 | 6 | **SLC38A2** | SNAT2 | 417807 | 1 |
| **GRM1** | mGluRs | 421614 | 3 | **SLC16A7** | MCT2 | 417815 | 1 |
| **GRM2** | mGluRs | 427575 | 12 | **SLC9A2** | NHE2 | 418719 | 1 |
| **GRM3** | mGluRs | 418644 | 1 | **SLC7A1** | CAT-1 | 418922 | 1 |
| **GRM5** | mGluRs | 373933 | 1 | **SLC2A5** | GLUT-5 | 419438 | 21 |
| **GRM7** | mGluRs | 416112 | 12 | **SLC7A7** | y+LAT1 | 420216 | 2 |
| **GRM8** | mGluRs | 427853 | 1 | **SLC9A3** | NHE3 | 420801 | 2 |
| **PKD2L1** | - | 428954 | 6 | **SLC9A3R1** | - | 422108 | 18 |
| **HCN3** | - | 100859704 | 25 | **SLC7A2** | CAT-2 | 422730 | 4 |
| **HCN1** | - | 431590 | Z | **SLC1A2** | EAAT2 | 423156 | 5 |
| **HCN2** | - | 770384/428335 | 28 | **SLC1A1** | EAAT-3 | 427352 | Z |
| **HCN4** | - | 427478 | 10 | **SLC3A1** | SLC1A5, ASCT2, rBAT | 428569 | 3 |
| **AQP7** | aquaporin 7 | 426892 | Z | **SLC7A11** | xCT | 428731 | 4 |
| **AQP2** | aquaporin 2 | 431304 | 33 | **SLC36A1** | PAT1 | 770250 | 13 |
| **AQP5** | aquaporin 5 | 431305 | 33 | **BSG** | CD147 | 770363 | 28 |
| **AQP3** | - | 426894 | Z | **SLC5A10** | SGLT5 | 770581 | 14 |
| **AQP1** | - | 420384 | 2 | **SLC6A14** | ATB0,+ | 10085752 | 4 |
| **AQP4** | - | 421088 | 2 | **SLC16A3** | - | 395383 | 18 |
| **AQP8** | - | 416566 | 14 | **SLC38A1** | - | 417806 | 1 |
| **AQP9** | - | 415402 | 10 | **SLC1A2** | - | 423156 | 5 |
| **AQP10** | - | 101749384 | 25 | **SLC2A4RG** | GEF | 100858363 | 20 |
| **AQP11** | - | 426725 | 1 | **Gust** | GNAT3 | 427851 | 1 |
| **AQP12A** | - | 424861 | 9 | **AGXT2** | - | 431666 | Z |
| **T2R1** | TAS2R1; TAS2R40 | 101749182 | 1 | **DLAT** | - | 419796 | 24 |
| **T2R2** | TAS2R2; TAS2R4 | 101747503 | 1 | **PDHB** | - | 416066 | 12 |
| **T2R7** | TAS2R7; TAS2R3 | 771663 | 3 | **PDHX** | - | 423154 | 5 |
| **GNAT3** | Gustducin | 427851 | 1 | **SLC2A2** | - | 396272 | 9 |
|  | SCNN1G | 416579 | 14 | **GLUT4** | - | 100858363 | 20 |
| **ENaC** | SCNN1B | 427673 | 14 | **YWHAE** | - | 417554 | 19 |
|  | SCNN1D | 428184 | 21 | **YWHAQ** | - | 421932 | 3 |
|  | SCNN1A | 396050 | 1 | **YWHAZ** | - | 425619 | 2 |
| **GPRC5B** |  | 101747453 | 14 | **ENO1** | - | 396017 | 21 |
| **SLC15A1** | PepT-1 | 378789 | 1 | **PKM** | - | 396456 | 10 |
| **SLC2A8** | GLUT8 | 378802 | 17 | **ALT** | GPT2 | 415746 | 11 |
| **SLC16A3** | MCT4 | 395383 | 18 | **OGDH** | - | 426429 | 22 |
| **ANPEP** | APN | 395667 | 10 | **CS** | - | 100858903 | 33 |
| **SLC16A8** | MCT3 | 396041 | 1 | **LIAS** | - | 422791 | 4 |
| **SLC2A1** | SLC2A1, GLUT1 | 396130 | 21 |  |  |  |  |

**Table S3.** Performance parameters of broiler chickens in the double choice (DC) experiments of feed vs feed supplemented with amino acid (AA) during d 28-42 (week 5 and 6). **FG**: fast-growing chickens; **SG**: slow-growing chickens; **T1**: control feed; **T2**: essential amino acid (Met, Lys, Thr); **T3**: non-essential amino acid (Ala, Asp, Asn); **G×T**: group × treatment interaction. ^a, b^ means not sharing common letters are significantly different.

| **DC test** | **Effect** | **Body weight (g)** | | | **Weight gain (g)** | **Feed intake (g)** | **FCR(g:g)** |
| --- | --- | --- | --- | --- | --- | --- | --- |
|  |  | **d 28** | **d 35** | **d 42** | **d 28-42** | **d 28-42** | **d 28-42** |
| Feed vs Feed Supplemented with AA | Main effect |  |  |  |  |  |  |
|  | Group |  |  |  |  |  |  |
|  | FG | 1900^a^ | 2596^a^ | 3465^a^ | 1565^a^ | 3019^a^ | 1.94 |
|  | SG | 1347^b^ | 1935^b^ | 2664^b^ | 1317^b^ | 2603^b^ | 1.99 |
|  | Treatment |  |  |  |  |  |  |
|  | T1 | 1645 | 2281 | 3098 | 1453 | 2857 | 1.99 |
|  | T2 | 1584 | 2218 | 2981 | 1399 | 2667 | 1.91 |
|  | T3 | 1641 | 2298 | 3112 | 1471 | 2910 | 1.99 |
|  |  |  |  |  |  |  |  |
|  | P-value |  |  |  |  |  |  |
|  | Group | <0.001 | <0.001 | <0.001 | <0.001 | <0.001 | NS |
|  | Treatment | NS | NS | NS | NS | NS | NS |
|  | G×T | NS | NS | NS | NS | NS | NS |

**Table S4**. Gene expression levels for amino acid sensor/transporters in fast- (**FG**) compared to slow-growing (**SG**) chickens using both RNAseq and qPCR methods. **T1R1/T1R3**: Umami taste receptors; **GPR92** and **139**: G protein coupled receptor 92 and 139; **CaSR**: Ca sensing receptor, **Gust**: α-gustducin, **B-ACT**: Beta actin, and **GAPDH**: Glyceraldehyde 3-phosphate dehydrogenase.

| Analysis | Gene | SG | FG | P value |
| --- | --- | --- | --- | --- |
| RNAseq | T1R3 | 0.107±0.022 | 0.109±0.005 | 0.944 |
|  | **T1R1** | **0.729±0.044** | **0.924±0.094** | **0.098** |
|  | **Gust** | **0.237±0.067** | **0.307±0.029** | **0.009** |
|  | GPR92 | 0.202±0.086 | 0.243±0.086 | 0.743 |
|  | GPR139 | 0.057±0.019 | 0.062±0.019 | 0.841 |
|  | CasR | 0.450±0.075 | 0.458±0.046 | 0.924 |
|  | GAPDH | 379.55±17.87 | 413.39±19.23 | 0.245 |
|  | B-ACT | 925.40±71.26 | 988.15±36.21 | 0.407 |
| qPCR | T1R3 | 29.93±0.200 | 29.61±0.116 | 0.763 |
|  | **T1R1** | **28.15±0.162** | **28.54±0.165** | **0.087** |
|  | **Gust** | **27.91±0.199** | **28.90±0.430** | **0.021** |
|  | GPR92 | 26.53±0.193 | 26.87±0.226 | 0.155 |
|  | GPR139 | 30.70±0.486 | 30.91±0.308 | 0.302 |
|  | CasR | 27.69±0.210 | 27.86±0.110 | 0.271 |
|  | GAPDH | 16.57±0.162 | 16.58±0.127 | 0.411 |
|  | B-ACT | 15.54±0.172 | 15.56±0.138 | 0.493 |

**References for the Supplementary Material**

Niknafs, S. Fortes, M. Roura, E. 2018. Expression of specific amino acid receptors in the oral cavity is associated with growth rate and feed intake in broiler chickens. World Congress on Genetics Applied to Livestock Production, Auckland, New Zealand

Pfaffl, M.W., 2001. A new mathematical model for relative quantification in real-time RT–PCR. Nucleic Acids Research 29, e45-e45.

Ruijter, J.M., Ramakers, C., Hoogaars, W.M.H., Karlen, Y., Bakker, O., van den Hoff, M.J.B., Moorman, A.F.M., 2009. Amplification efficiency: linking baseline and bias in the analysis of quantitative PCR data. Nucleic Acids Research 37, e45-e45.
